# Exploring the potential of IL-10 for risk assessment and early intervention in pediatric ALL

**DOI:** 10.1186/s12885-024-12677-w

**Published:** 2024-08-08

**Authors:** Roqaia E. Radwan, Ahmad Darwish, Afaf M. Elsaid, Wafaa M. El-kholy

**Affiliations:** 1 Physiology Section, Zoology Department, Faculty of Science, Mansoura University, Mansoura, Egypt; 2 Hematology, Oncology and Bone Marrow Transplantation Unit, Pediatric Department, Faculty of Medicine, Mansoura University, Mansoura, Egypt; 3 Genetics Unit, Children Hospital, Mansoura University, Mansoura, Egypt

**Keywords:** ALL, IL10, Gene polymorphism, Cytokine levels

## Abstract

Acute lymphoblastic leukemia (ALL), a leading cause of childhood cancer, targets immune system B and T cells. While understanding its causes is crucial, predicting susceptibility holds immense power for early diagnosis and intervention. This study explored the potential of interleukin 10 (IL-10), a key immune regulator, as a predictive tool in Egyptian children. Investigating 100 ALL patients and 100 healthy controls, we analyzed the *IL10* gene polymorphism (-1082 A/G) and serum levels. Strikingly, both the G allele and higher serum IL-10 levels were significantly associated with increased ALL risk (p < 0.05, OR > 1). Moreover, IL-10 emerged as a remarkably accurate predictor, boasting an AUC of 0.995, with a sensitivity of 97% and specificity of 96%. These findings unveil the potential of IL-10 as a powerful predictive tool for pediatric ALL in the studied Egyptian population. Identifying individuals with the GG/AG haplotype and elevated IL-10 levels could enable early intervention and potentially improve outcomes. While further validation in larger and more diverse populations is needed, this study paves the way for personalized risk assessment and potentially revolutionizes how we combat this childhood killer.

**Supplementary Information:**

The online version contains supplementary material available at 10.1186/s12885-024-12677-w.

## Introduction

Acute lymphocytic leukemia (ALL) is a B or T lymphocyte precursor cell cancer. These cells are immature and proliferate (grow and divide) uncontrollably. This leads to replacing normal bone marrow and lymphoid organ cells with abnormal lymphocytes. ALL accounts for approximately 2% of all lymphoid neoplasms diagnosed in the United States [1]. Unfortunately, 4,000 children in the United States each year are diagnosed with ALL, with the majority of cases occurring in children under the age of 18. This makes ALL the most common childhood malignancies. The peak age of diagnosis is between 2 and 10 years of age [2].

Accurate risk group stratification is crucial for effective therapy in ALL. Modern algorithms use binary categories based on clinically relevant risk factors, including white blood cell count and minimal residual disease. The Children’s Oncology Group (COG) B-ALL algorithm integrates various clinical and genetic risk markers and MRD assessments [3].

Prognostic models in oncology guide clinical decisions by adjusting treatment intensity based on individual relapse risk. Effective models must undergo stringent quality control, including thorough internal and external validation [4].

Future research should refine the PICOG model by incorporating new prognostic factors. Modern prediction models must adapt to new discoveries, such as high-throughput sequencing for MRD and new genetic markers. Model-derived risk scores like PICOG can integrate new information more readily than traditional algorithms, which require extensive clinical knowledge for new markers. Established statistical methods allow for incorporating new data, potentially improving the model's ability to distinguish outcomes and enabling targeted research [5].

The molecular pathogenesis of ALL involves a complex interplay of genetic and epigenetic alterations, including abnormal gene expression, which dysregulate key cellular signaling pathways and lead to uncontrolled proliferation and survival of neoplastic cells. Established risk factors for B-ALL include prior parvovirus infection, high birth weight, and exposure to environmental toxins, which can induce DNA methylation and other chromosomal aberrations [6].

Genetic predispositions are critical in understanding ALL. Chromosomal abnormalities and genetic mutations affecting lymphoid precursor cells are hallmark features of the disease. Children with genetic syndromes like Down syndrome, constitutional mismatch repair deficiency, Fanconi anemia, Bloom syndrome, ataxia telangiectasia, neurofibromatosis, and Nijmegen breakage syndrome have a significantly higher risk of developing ALL due to defects in DNA repair and cell division processes. Notably, individuals with Down syndrome face a 20-fold increased risk, with distinct disease characteristics and poorer prognosis compared to the general population. Inherited genetic variants, particularly those in genes frequently targeted by somatic mutations in ALL, further underscore the complex interplay between genetic susceptibility and disease biology [7–11].

### ALL identification

So far, immunophenotyping is the only way to identify many of the diverse of ALL entities that exist. This leads scientists towards modern cytogenetic and molecular biology techniques to detect specific chromosomal rearrangements and genetic alterations. The specific ALL etiology is not well-known, but detecting predictors of the risk of ALL has more impact on the management of the disease. Up till now, the management has not been initiated except after symptoms appeared which are often nonspecific, therefore the diagnosis is made based on a pathological, immunochemical, and molecular evaluation of the bone marrow aspirate and biopsy material, with the criterion of at least 20% of bone marrow lymphoblasts for the definitive ALL diagnosis. Such procedures of diagnosis will lead to reaching the latest stages of the disease which it difficult to treat. Hence, ultimately this may cause a higher mortality rate.

Due to the new classification of ALL which takes into account the clinical diversity and cytogenetics of different ALL entities, it has dramatically changed the management strategies and improved prognosis for patients with ALL subtypes. This finally led to the emergence of novel targeted therapeutics [12]. Our study has gone towards detecting predictors of the risk which can allow predicting the susceptibility in the pediatrics.

Moreover, it is encouraging to say that recent studies have contributed to understanding the genetic basis of clonal evolution, relapse, and the role of inherited genetic variants in leukemogenesis. This means that many of these findings are implementing clinical sequencing in the management of leukemia and are expected to improve diagnosis, monitoring of residual disease, and early detection of relapse and to guide precise therapies [13].

While inflammation acts as the initial signaling pathway of the immune response, the IL-10acts as anti-inflammatory cytokines in the body which interrupts the response of the immune system. It is secreted by various immune cells including monocytes, macrophages, dendritic cells, Lymphocytes, granulocytes, epithelial cells, keratinocytes, and mast cells. It is important to say that what makes IL-10 the key objective of the study is that it limits the secretion of pro-inflammatory cytokines and controls the differentiation and proliferation of macrophages, T cells, and B cells the two lineages of ALL. Mutations in *IL 10* or components of its signaling pathway that reduce or abolish their anti-inflammatory properties were believed to be associated with the pathogenesis of hyperinflammatory disorders which are symptoms of cancers [14].

### ALL treatment

Unveiling leukemia's hidden presence early is paramount to the success of treatment and the extension of life. However, current screening methods often fall short, plagued by low sensitivity and specificity, hefty costs, and underwhelming participation rates. A fresh approach, harnessing novel and innovative biomarkers from peripheral blood, holds promise as a more convenient and appealing alternative, potentially boosting participation and facilitating earlier diagnosis [15].

This research aimed to investigate the potential of biomarkers, particularly IL-10, as susceptibility risk factor for ALL in pediatric populations. Building upon previous studies that have highlighted the limitations of current screening methods, this study sought to explore and validate the utility of these biomarkers to significantly improve the assessment of ALL susceptibility. Existing screening approaches have demonstrated inadequacies, often resulting in missed cases or unnecessary investigations. Leveraging the promising performance of IL-10, which has shown high sensitivity and specificity in preliminary analyses, we aim to provide a comprehensive understanding of its role in ALL susceptibility. The overarching goal was to contribute valuable insights that could potentially transform the landscape of ALL risk assessment, addressing current challenges and paving the way for improved evaluation methods.

The implications of successful findings in this research are substantial. If IL-10 proves to be a reliable indicator of ALL susceptibility, the clinical applications could be transformative. Integrating these biomarkers into routine screening protocols could facilitate earlier and more accurate identification of at-risk individuals, reducing the incidence of missed cases and unnecessary medical interventions. Furthermore, the potential for non-invasive and highly reliable tools holds significant promise for improving patient outcomes through timely intervention and personalized treatment strategies.

In summary, this research aimed to validate and establish the efficacy of IL-10 as a susceptibility marker for ALL, with the ultimate goal of contributing to the development of more precise and efficient risk assessment methods. The anticipated outcomes include addressing current diagnostic challenges and paving the way for early intervention and improved prognosis for individuals at risk of ALL.

## Methods

### Healthy controls and leukemia patients

The present study was conducted on ALL patients and healthy controls. The ALL group includes 100 newly diagnosed individuals ranging from 2– 17 years old (median 9.5; 64 males and 36 females) and 100 healthy individuals (40 males and 60 females) with age ranging from 2– 17 years old (median 9.5 were selected as a healthy control group. They were unrelated to ALL patients, had the same ethnicity, and were living in Dakahlia Government—Egypt.

### Study setting

The ALL patients were recruited from the Children's hospital and oncology center of Mansoura University—Egypt. In the healthy control, samples were collected from blood donors and from children attending outpatient clinics for a routine checkup.

### Deadline of the study

From March 2021 to March 2022.

### Inclusion criteria

The patient cohort for this study will comprise 100 individuals newly diagnosed with ALL, specifically targeting patients aged below 18 years. Inclusion is open to both male and female participants, with a weight range between 12 and 60 kg.

### Exclusion criteria

Exclusion criteria for this study involve individuals who have undergone prior treatments such as chemotherapy or radiotherapy, and those with pre-existing chronic illnesses, both in the patient and healthy groups.

### Inclusion criteria of healthy group

The healthy control group will comprise 100 individuals with no history of ALL or any other type of leukemia. Age will be matched to the patient group, specifically targeting individuals below 18 years old. Inclusion is open to both male and female participants. Weight range should ideally be similar to the patient group, between 12 and 60 kg (although slight variations might be acceptable depending on the study design).

### Exclusion criteria of healthy group

Individuals with any pre-existing chronic illnesses will be excluded. This aligns with the exclusion criteria for the patient group to ensure a fair comparison. Additionally, to minimize confounding factors, individuals who have received blood transfusions or immunocompromising medications in the recent past (within a timeframe you define) will be excluded.

ALL was diagnosed by cytomorphological, cytochemical, and immunophenotyping methods. ALL new cases without previous therapy were included in this study. Patients had a full medical history as well as general and local clinical examination. The clinical data of ALL patients were collected from the patient's archive.

Leukemia-associated immunophenotypes were studied at diagnosis by using the following antibodies: CD117, CD33, CD13, MPO, CD3, CD7, CD10, CD19, and CD22.

### Flow cytometry immunophenotyping

Leukemia-associated immunophenotypes were studied using flow cytometry on whole peripheral blood (PB). The process involved staining with antibodies CD117, CD33, CD13, MoAb antimyeloperoxidase {MPO}, CD3, CD7, CD10, CD19, and CD22, all sourced from Becton Dickinson Immunocytometry Systems (San Jose, CA). The cells were incubated with these monoclonal antibodies (MoAbs) and analyzed using a Fluorescence Activated Cell Analyzer (FACScan, San Jose, CA) device with Cell Quest software (Cell Quest™ Software, Becton Dickinson Immunocytometry Systems). The analysis parameters included acquiring 20,000 events per tube and assessing forward scatter (FSC) for cell size and side scatter (SSC) for cell complexity, with fluorescence in FL1 (FITC, green), FL2 (PE, orange), and FL3 (PerCP, red). Results were considered positive if at least 25% of cells reacted with the MoAbs.

### Antibody colors

CD117: PE (Phycoerythrin), CD33: FITC (Fluorescein isothiocyanate), CD13: APC (Allophycocyanin), MPO: PerCP (Peridinin-Chlorophyll-Protein), CD3: PE-Cy7 (Phycoerythrin–Cyanine 7), CD7: FITC, CD10: APC, CD19: PerCP and CD22: PE

### Sampling

2 mL of venous blood was collected and placed on EDTA for both ALL patients and the control group. Whole blood for the investigation of PCR of *IL10* gene polymorphism and plasma serum for the estimation of anti-inflammatory cytokine IL-10 levels.

### Typing of *IL10* gene polymorphism

DNA was isolated according to [16]. The Gene Jet TM purification kit (Genomic DNA purification Kit, USA) was used for DNA extraction. Electrophoresis through a 1% agarose slab gel was used to analyze DNA samples. Agarose gel electrophoresis is used to separate DNA fragments based on their molecular weight, with smaller fragments moving faster than larger fragments. The DNA was stained with ethidium bromide to be visualized and photographed by a digital camera. The PCR method is based on the enzymatic amplification of a fragment of DNA flanked by two primers (short oligonucleotides) that hybridize to opposite strands of the target sequence and primer extended according to the complementary sequence using the DNA polymerase enzyme. The genotypes of the *IL10* SNPs were discriminated by Tetra-Primer Amplification Refractory Mutation System-Polymerase Chain Reaction method (Tetra-Primer ARMS-PCR). This method involves a single PCR with no post-PCR manipulation. Computer software program for primers design has been developed by Ye et al., [17]. The primers of the reaction were designed, using the program default settings, to amplify the genomic DNA fragments containing the two SNPs at positions -1082(G ⁄ A), as shown in Table 1.

**Table 1:** Information of Tetra-Primer ARMS-PCR primers used

**IL-10 SNP**	**Primer Sequence**	Tm (C) AT (C)	Product size (bp)
-1082 G > A	Forward inner primer (A allele): 5¢-AACACTACTAAGGCTTCTTTGGGCAA	64 62	A allele: 197
	Reverse inner primer (G allele): 5¢-ACTTTCCTCTTACCTATCCCTACTTCACC	64	G allele: 288
	Forward outer primer: 5¢-CCAGTTACAGTCTAAACTGGAATGCAG	64	430 (from two
	Reverse outer primer: 5¢-CTTGGATTAAATTGGCCTTAGAGTTTCT	64	outer primers)

The DNA was stained with ethidium bromide to be visualized and photographed by a digital camera.

### Estimation of serum levels of (IL-10)

The assay of IL-10 serum levels was determined according to [18]. A specialized kit (Boster’s Human IL-10 ELISA Kit) was used to measure serum levels with an enzyme-linked immunosorbent assay (ELISA).The sensitivity of Boster’s Human IL-10 ELISA Kit is typically around < 10 pg/mL. The detection range is 15.6 pg/mL to 1000 pg/mL.

Standard venipuncture techniques have been used to draw blood samples and as quickly as possible serum was isolated from the blood. Then we permitted samples to clot for sixty minutes at 25°C, centrifuged for ten minutes (4°C) and serum extracted. -20°C was the degree of storage, keeping samples from of loss of contamination and bioactivity. Freeze–thaw cycles were avoided.

The preparation and execution of an ELISA was applied to measure human IL-10 levels in samples. It involved diluting samples and standards, preparing reagents, and then loading them onto a pre-coated microplate. After incubation and washing steps, a colorimetric reaction revealed the amount of IL-10 present, which is quantified by reading the absorbance at 450nm.

### Statistical analysis

The data was first revised, coded, and tabulated using SPSS software. (IBM Corp. Released 2017. IBM SPSS Statistics for Windows, Version 25.0. Armonk, NY: IBM Corp.). Different tests were then applied based on the data type and research question. For genetic data, allele frequency and Hardy–Weinberg equilibrium were assessed. Normality of numerical data was checked using the Kolmogorov–Smirnov test. Descriptive statistics like mean, median, and standard deviation were calculated. Further analysis involved t-tests, ANOVA, Mann–Whitney tests, Kruskal–Wallis tests, chi-square tests, Monte Carlo tests, and logistic regression depending on the variables and study objectives. Significance levels were set at *p* < 0.05 with 95% confidence intervals.

## Results

### Baseline studies

**Fig. 1:**
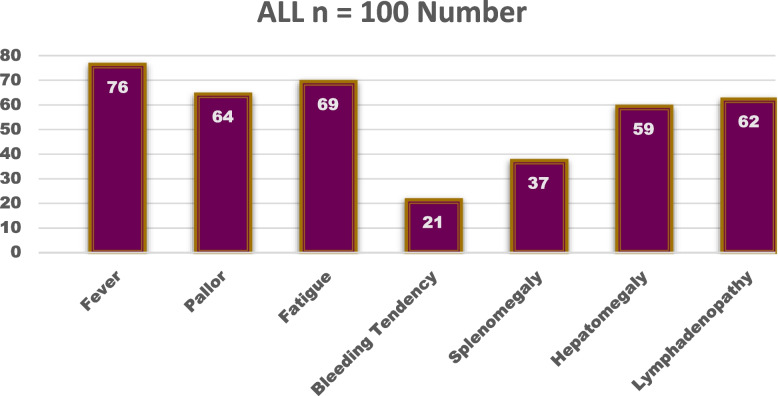
Clinical characteristics among patients with ALL

A study was conducted on a sample of 100 patients with acute lymphoblastic leukemia. The clinical characteristics of the patients were investigated, including fever, pallor, fatigue, bleeding tendency, splenomegaly, hepatomegaly, and lymphadenopathy. The table summarizes the distribution of these characteristics, with the number and percentage of patients presenting with each symptom as shown in Fig. 1.

### Genetic studies

A specific Single Nucleotide Polymorphism (SNP) is located within the Interleukin-10 (IL10) gene on chromosome 1. The SNP, designated rs1800896 by the National Center for Biotechnology Information (NCBI) [NCBI SNP database], involves two possible alleles: A (adenine) and G (guanine). The table indicates that the "reference allele," the more common version in the population, is A, while the "alternative allele" is G, as shown in Supplementary Table 1 (S1).

### Hardy Weinberg equilibrium for studied SNPs

The assessment of Hardy–Weinberg equilibrium for IL-10 genotypes was presented in two groups in Table 3: Control (*n* = 100) and ALL (*n* = 100). The observed and expected frequencies of IL10 -1082 A/G (rs1800896) genotypes are provided for both groups. In the control group, the observed frequencies for AA, AG, and GG genotypes were 50, 46, and 4, respectively, compared to the expected frequencies of 53.29, 39.42, and 7.29, respectively. Similarly, in the ALL group, the observed frequencies for AA, AG, and GG genotypes were 13, 40, and 47, respectively, compared to the expected frequencies of 10.89, 44.22, and 44.89, respectively. The calculated probabilities (P) for the Control group and ALL group were 0.248 and 0.634, respectively. This suggests that there is a deviation from the Hardy–Weinberg equilibrium for the IL10 -1082 A/G (rs1800896) genotypes, particularly in the Control group where the observed and expected frequencies differ noticeably, as shown in Table 2.

**Table 2:** Assessment of Hardy Weinberg equilibrium for IL10 genotypes

*Genetic polymorphism*		*Control n* = *100*		*ALL n* = *100*	
		***Observed***	***Expected***	***Observed***	***Expected***
***IL10 − 1082 A/G (rs1800896)***	***AA***	*50*	*53.29*	*13*	*10.89*
	***AG***	*46*	*39.42*	*40*	*44.22*
	***GG***	*4*	*7.29*	*47*	*44.89*
	***P***	*0.248*		*0.634*	

No significant differences were found between observed and expected counts in each group

### IL-10 levels among studied groups

**Fig. 2:**
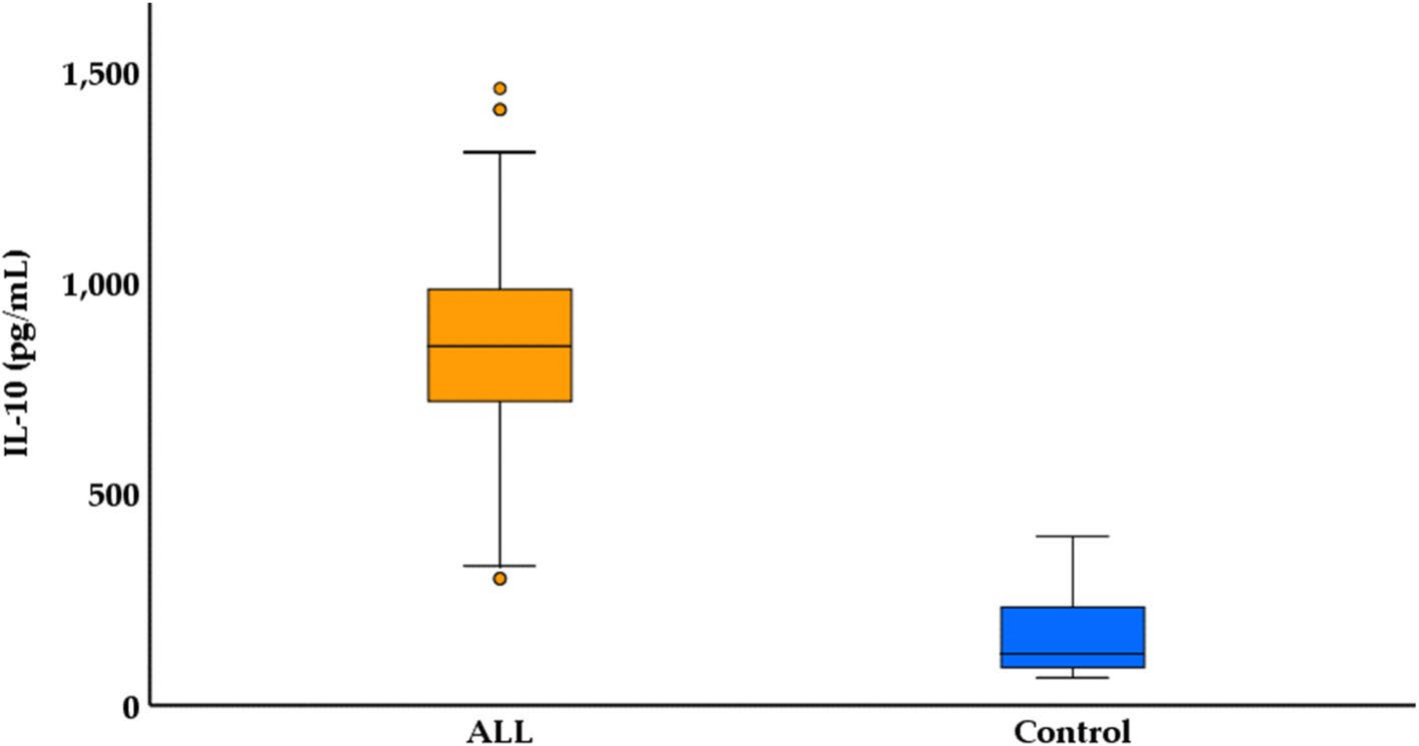
Boxplot for IL-10 among ALL patients and control group

The IL-10 levels were measured in pg/mL and were summarized by mean ± standard error (SE), median, and range. In the Control group, the mean IL-10 level was 172.91 pg/mL ± 9.87 SE, with a median of 122.0 pg/mL and a range of 65.40 pg/mL to 400.0 pg/mL. In contrast, the ALL group exhibited significantly higher IL-10 levels, with a mean of 833.78 pg/mL ± 25.01 SE, a median of 850.0 pg/mL, and a range of 300.0 pg/mL to 1460.0 pg/mL. The statistical test (U = 52.0, *p* < 0.001) indicates a significant difference in IL-10 levels between the two groups, highlighting an elevated IL-10 expression in ALL patients compared to the control group, as shown in Table 3 and Fig. 2.

**Table 3:** Comparison of IL-10 levels among ALL patients and control group

	***Control n* = *100***	***ALL n* = *100***	***Test (p)***
***IL-10 (pg/mL)***			
* Mean* ± *SE*	*172.91* ± *9.87*	*833.78* ± *25.01*	*U* = *52.0* *p* < *0.001*
* Median*	*122.0*	*850.0*	
* Range*	*65.40 – 400.0*	*300.0 – 1460.0*	

*SE* Standard error, *min* minimum, *max* maximum, *U* Mann Whitney test

### *IL10* − 1082 among studied groups

**Fig. 3:**
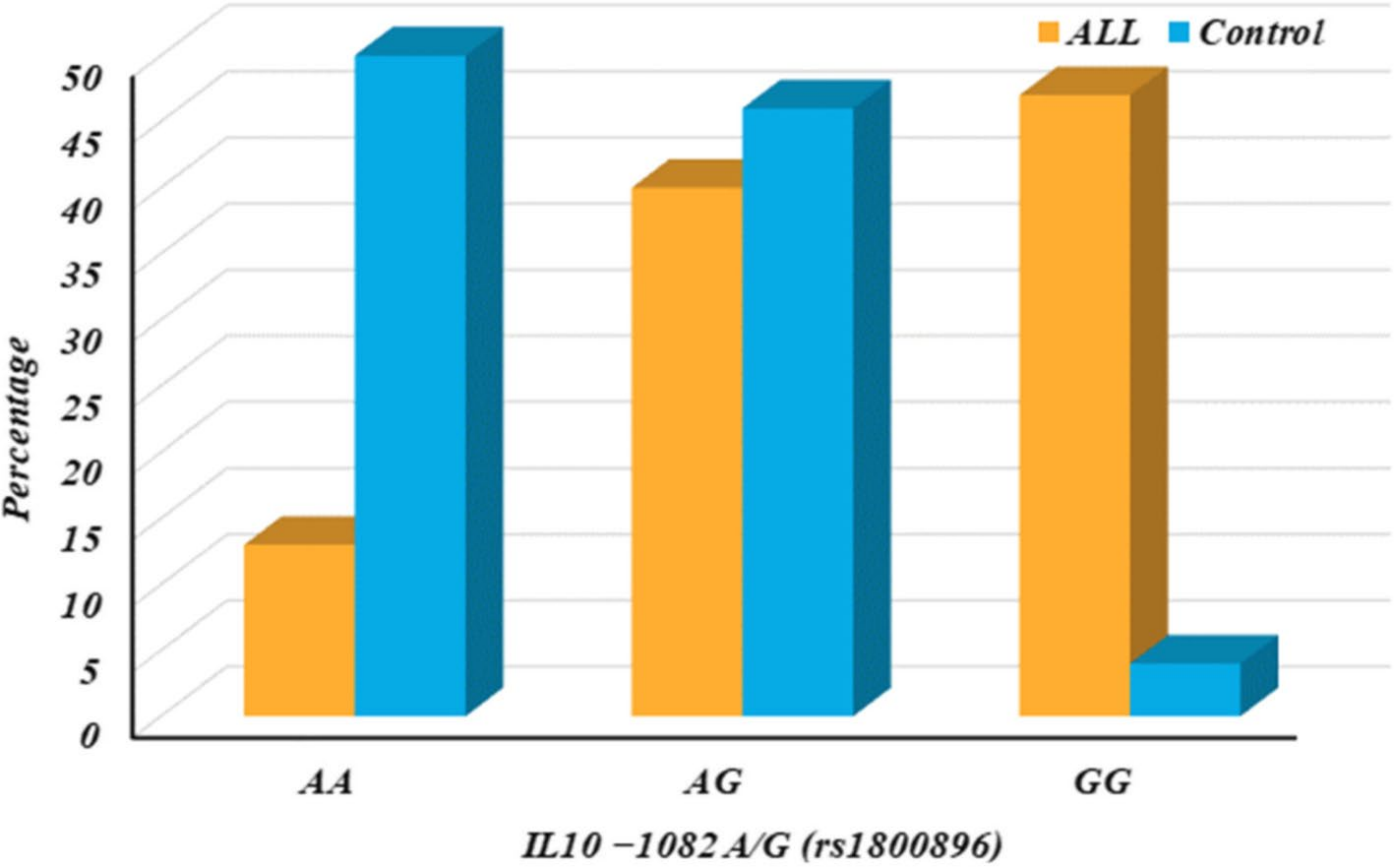
IL10 − 1082 A/G (rs1800896) among ALL patients and control group

The distribution of the *IL10* -1082 A/G (rs1800896) gene variant among 100 control subjects and a separate group of 100 individuals (ALL) were investigated in Table 5. It compares the allele frequencies (A and G) and genotypes (AA, AG, GG) between the two groups and reports the statistical significance (p-value) of these differences. Additionally, it calculates Odds Ratios (OR) with 95% Confidence Intervals (CI) to assess the association between the genotypes and potential risk factors, as shown in Table 4 and Fig. 3.

**Table 4:** IL10 − 1082 A/G (rs1800896) among ALL patients and control group

*IL10 − 1082 A/G (rs1800896)*		*Control n* = *100*		*ALL n* = *100*		*p - value*	*OR (95% CI)*
		*N.*	***%***	*N.*	***%***		
***Genotypes***	***AA***	*50*	*50.0*	*13*	*13.0*	*-*	*Reference*
	***AG***	*46*	*46.0*	*40*	*40.0*	*0.001*	2.078 (1.339–3.225*)*
	***GG***	*4*	*4.0*	*47*	*47.0*	< *0.001*	9.345 (5.059*–*17.262*)*
***Dominant model***	***AA***	*50*	*50.0*	*13*	*13.0*	*-*	*Reference*
	***AG* + *GG***	*50*	*50.0*	*87*	*87.0*	< *0.001*	3.204 (2.125*–*4.831*)*
***Recessive model***	***AA* + *AG***	*96*	*96.0*	*53*	*53.0*	*-*	*Reference*
	***GG***	*4*	*4.0*	*47*	*47.0*	< *0.001*	5.964 (3.460*–*10.279*)*
***Alleles***	***A***	*146*	*73.0*	*66*	*33.0*	*-*	*Reference*
	***G***	*54*	*27.0*	*134*	*67.0*	< *0.001*	2.868 (2.213*–*3.716*)*

*N* Number, *OR* Odds ratio, *CI* Confidence interval, Reference, according to NCBI database; *A* Adenine, *G *Guanine, *P* < 0.05 is considered significant; OR < 1 is considered protective; OR > 1 is considered risky

The key findings as shown in Table 4 & Fig. 3:

The G allele is significantly more frequent in the ALL group compared to controls (*p*-value < 0.001), suggesting a potential link between the G allele and the condition being studied in the ALL group.

Individuals with either AG or GG genotypes (carriers of the G allele) are more likely to be in the ALL group compared to those with only AA genotypes (*p*-value < 0.001). This is reflected in the elevated ORs for both dominant and recessive models. Overall, the data suggests a significant association between the IL10 -1082 A/G variant and the condition being investigated in the ALL group.

**Fig. 4:**
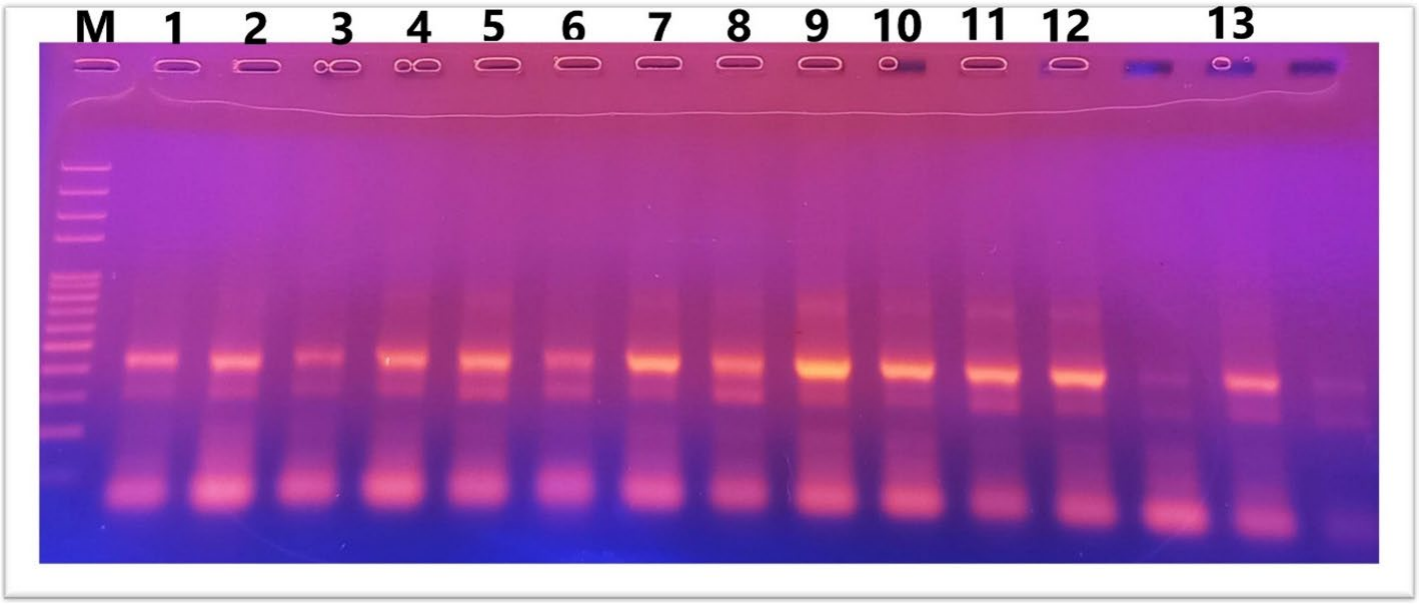
Lane 2,4,5,6,8,11.13 AG, Lane 1,3,7,9,10,12 GG where G allele at 288bp and A allele at 197 bp. 430 bp as internal control

**Fig. 5:**
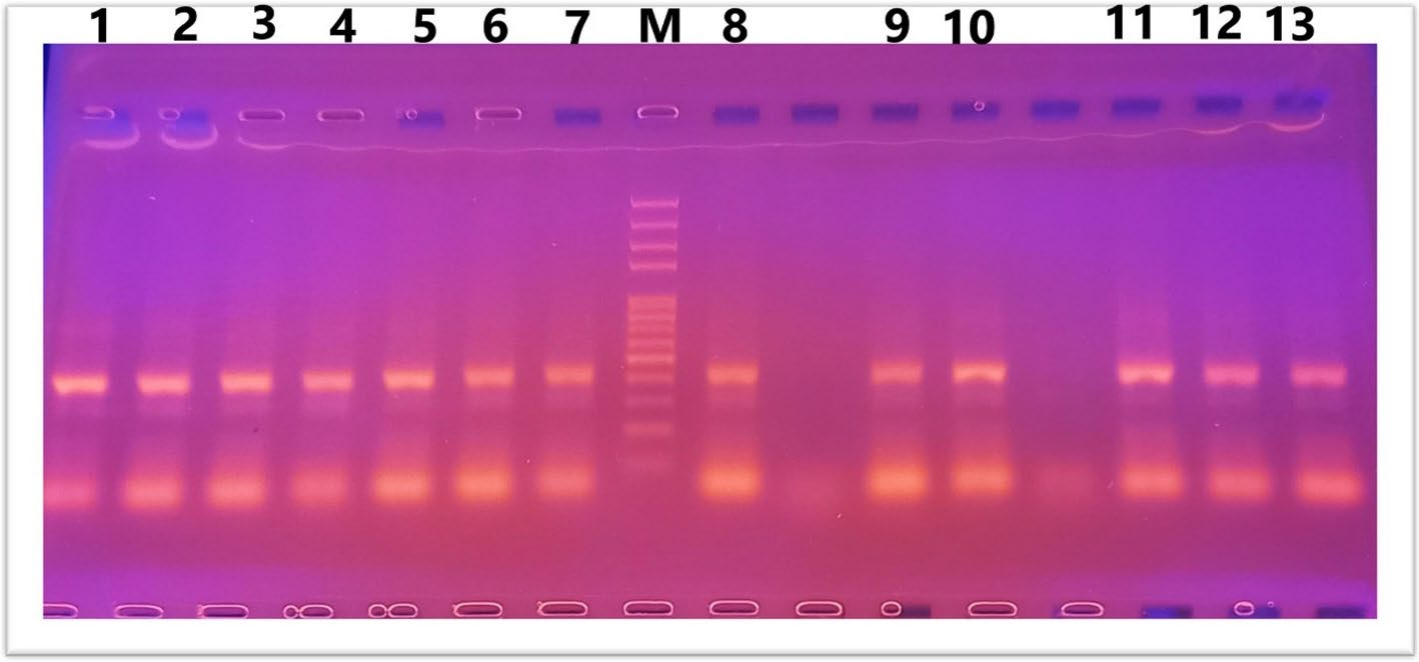
Lane 1,2,3,4,5,6,7,8,9,10,11,12,13 GG where G allele at 288bp

IL10 gene polymorphism (− 1082 A/G) was investigated using RFLP-PCR and classified as wild-type (AA), heterozygous carrier (AG), and homozygous variant (GG) genotypes. Results of PCR after gel electrophoresis are shown in Figs. 4 and 5.

Figures 4 and 5. Agarose gel electrophoresis for IL10 genotypes: ladder size marker (M) 50–1000 bp.

### Association of IL10 − 1082 with other parameters

#### Association between IL10 − 1082 A/G (rs1800896) with clinical presentation among ALL patients

The association between the IL10 -1082 A/G (rs1800896) gene variant and specific clinical presentations in the group of 100 ALL patients was analyzed in Supplementary Table 2 (S2). It divides the patients based on their genotypes (AA, AG, GG) and compares the frequency of various clinical symptoms like fever, pallor, fatigue, and bleeding tendency. Statistical tests (Chi-square, p-value) are used to assess the significance of these associations.

The results reveal significant associations between the genotype and presence of fever (*p*-value = 0.008) and pallor (*p*-value = 0.038). Patients with GG genotype (both G alleles) showed a higher prevalence of fever (78.7%) and pallor (61.7%) compared to those with AA genotype (no G alleles). However, no significant associations were found for fatigue (*p*-value = 0.368) or bleeding tendency (*p*-value = 0.379) across the genotypes.

These findings suggest that the IL10 -1082 A/G variant might influence the development of certain clinical symptoms in ALL patients, with the G allele potentially linked to an increased risk of fever and pallor as shown in Supplementary Table 2 (S2).

#### Association between IL10 − 1082 A/G (rs1800896) with organomegaly among ALL patients

The association between IL10 − 1082 A/G (rs1800896) genotypes and organomegaly among ALL (Acute Lymphoblastic Leukemia) patients was illustrated in Supplementary Table 3 (S3). The genotypes AA (*n* = 13), AG (n = 40), and GG (*n* = 47) are compared regarding their association with splenomegaly, hepatomegaly, and lymphadenopathy.

Splenomegaly showed a significant difference among the rs1800896 genotypes, with the highest incidence observed in the AG genotype (50.0%), followed by GG (31.9%) and AA (15.4%) genotypes (χ^2^ = 6.312, *p* = 0.043). Similarly, lymphadenopathy exhibited a significant difference among the genotypes, with the highest incidence associated with the AA genotype (92.3%), followed by AG (57.5%) and GG (57.4%) genotypes (χ^2^ = 7.104, *p* = 0.029).

On the other hand, hepatomegaly did not show a significant association with rs1800896 genotypes (χ^2^ = 2.155, *p* = 0.340), indicating that this particular genotype does not influence the presence of hepatomegaly among ALL patients.

Overall, these findings underscore the importance of genetic variations, specifically the rs1800896 genotypes, in influencing the development of specific organomegaly manifestations in ALL patients. Further studies with larger sample sizes and comprehensive genetic profiling may provide deeper insights into the genetic factors contributing to organomegaly in ALL and aid in developing targeted interventions for affected individuals.

#### Association between IL10 − 1082 A/G (rs1800896) with CRP among ALL patients

The serologic data for C-reactive protein (CRP) among 100 patients diagnosed with ALL were provided in Supplementary Table 4 (S4). It reveals that 61 patients (61%) tested negative for CRP, indicating normal or low inflammatory levels. The remaining 39 patients (39%) tested positive for CRP, suggesting potential inflammation or infection.

The association between IL10 − 1082 A/G (rs1800896) genotypes and CRP (C-reactive protein) levels among ALL patients was presented in Supplementary Table 5 (S5). The genotypes AA (n = 13), AG (*n* = 40), and GG (*n* = 47) are compared regarding their association with CRP levels categorized as negative or positive.

**Fig. 6:**
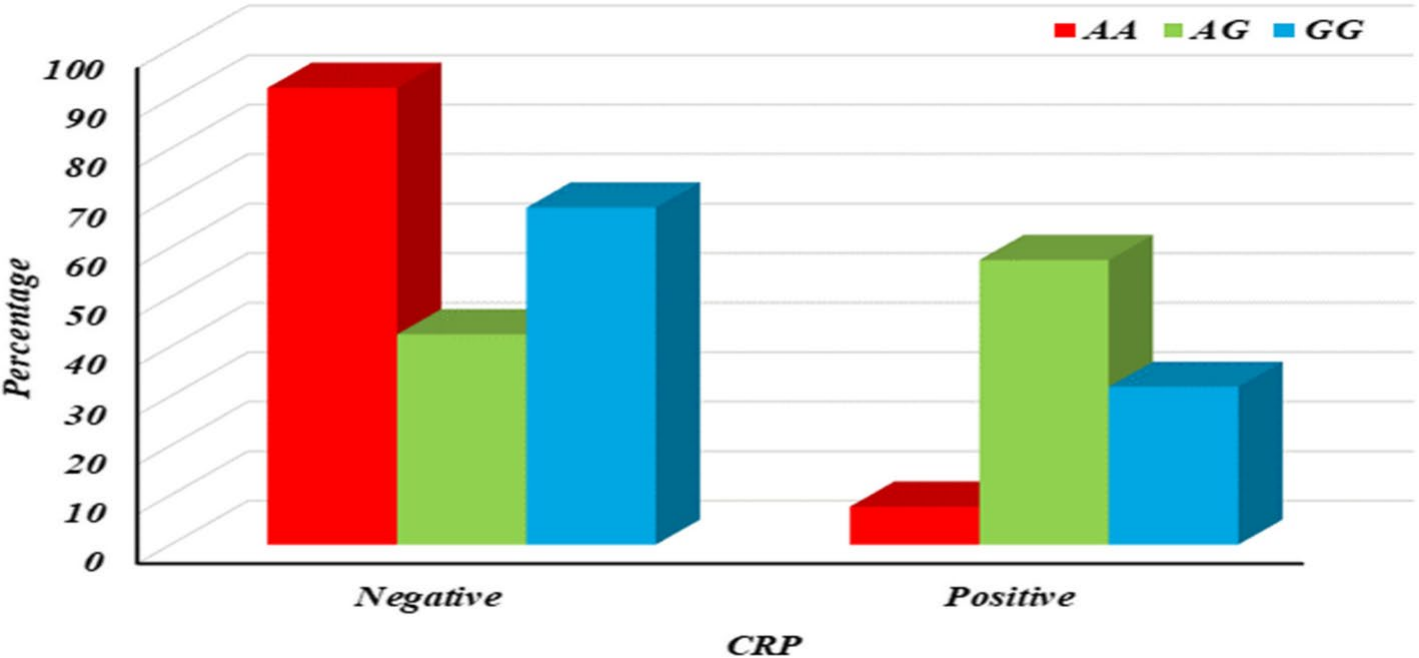
Column chart for association between IL10 − 1082 A/G (rs1800896) with CRP among ALL patients

The table displays the association between IL10 − 1082 A/G (rs1800896) genotypes and CRP levels among ALL patients, revealing a significant difference in CRP levels based on genotype (p1 = 0.002). Specifically, the AG genotype shows a notable association with elevated CRP levels compared to the AA genotype (p2 = 0.002) and the GG genotype (p4 = 0.016), while no significant difference is observed between AA and GG genotypes (p3 = 0.153). These findings suggest that the AG genotype may contribute to increased inflammation, as indicated by higher CRP levels, in ALL patients compared to the other genotypes, as shown in S5 & Fig. 6. This underscores the potential role of genetic variations in influencing inflammatory markers and highlights avenues for further research into personalized treatment strategies targeting inflammation in ALL.

The distribution of major subtypes in a population of ALL patients was depicted in Supplementary Fig. 3 (S3). B-cell ALL is the dominant subtype, accounting for 82% of the cases, while T-cell ALL makes up the remaining 18%.

**Fig. 7:**
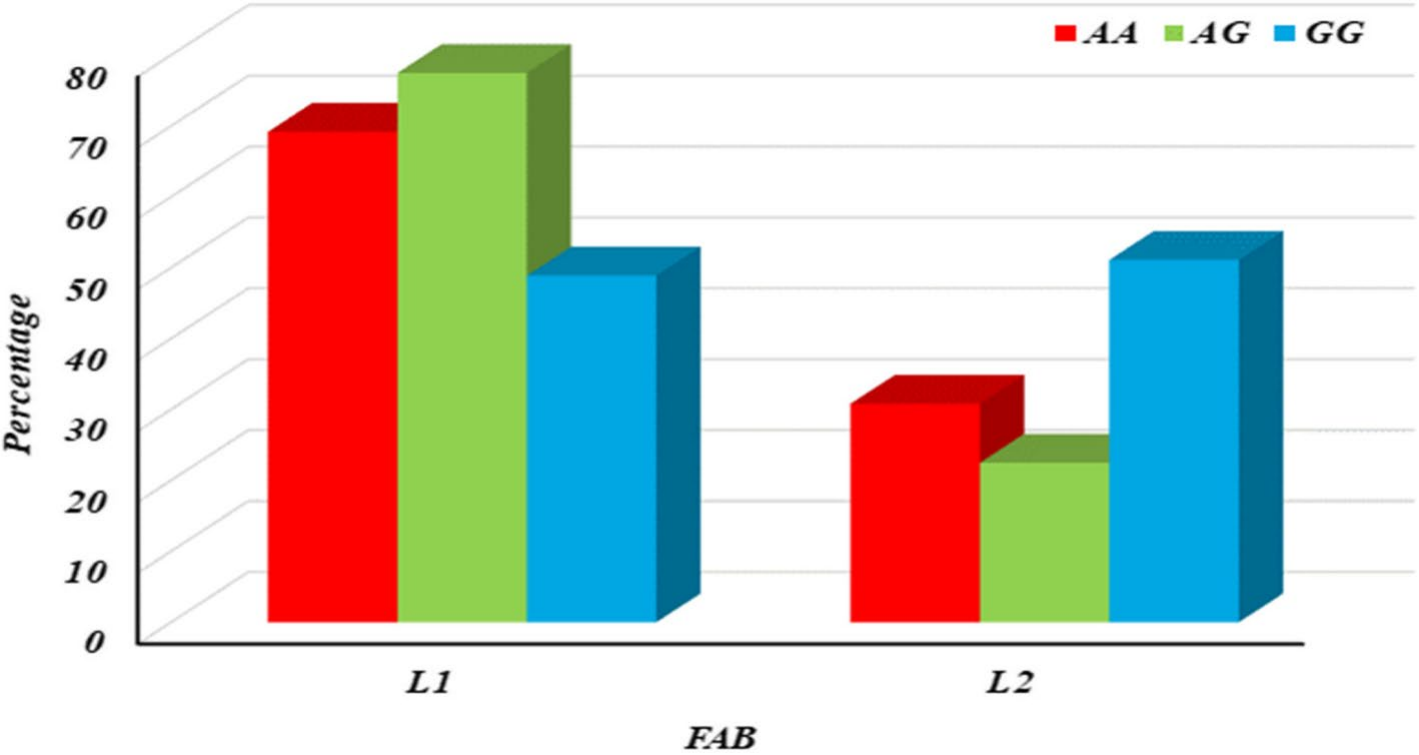
Column chart for association between IL10 − 1082 A/G (rs1800896) with FAB among ALL patients

The association between IL10 − 1082 A/G (rs1800896) genotypes and FAB (French-American-British) classifications among ALL patients was presented in Supplementary Table 6 (S6). The genotypes AA (*n* = 13), AG (*n* = 40), and GG (*n* = 47) are compared concerning their association with FAB subtypes L1 and L2. The FAB classification differed significantly among the rs1800896 genotypes (p1 = 0.020), primarily attributed to the significant association of L1 subtype with the AG genotype compared to GG (p4 = 0.006), indicating a potential link between the AG genotype and the L1 FAB subtype. Additionally, the L2 subtype showed a significant association with the GG genotype compared to AG (p4 = 0.006), suggesting a distinct genetic influence on FAB subtypes within ALL patients, as shown in S6 & Fig. 7.

### Prediction of ALL susceptibility

The regression analysis results for predicting susceptibility to ALL, both in univariable and multivariable models are presented in Table 5. In the univariable analysis, IL10 (*p* < 0.001, OR = 1.016, 95% CI = 1.008–1.024) and IL10 − 1082 (*p* < 0.001, OR = 3.204, 95% CI = 2.125–4.831) show significant associations with ALL susceptibility. When considering multiple variables in the multivariable analysis, IL10 remains significantly associated with ALL (*p* < 0.001, OR = 1.012, 95% CI = 1.002–1.054), indicating its independent predictive value. Additionally, IL10 − 1082 maintains a significant association (*p* = 0.006, OR = 1.058, 95% CI = 1.016–1.101) even after adjusting for other variables, suggesting its potential role as a predictive marker for ALL susceptibility.

**Table 5:** Regression analysis for prediction of ALL susceptibility

	**Univariable**			**Multivariable**		
	**P**	**OR**	**95% CI**	**p**	**OR**	**95% CI**
**IL10**	< *0.001*	1.016	1.008**–**1.024	< *0.001*	1.012	1.002**–**1.054
***IL10 − 1082***	< *0.001*	3.204	2.125**–**4.831	0.006	1.058	1.016**–**1.101

*OR* Odds ratio, *CI *Confidence interval. Logistic regression analysis was used

### Validity of IL10

**Fig. 8:**
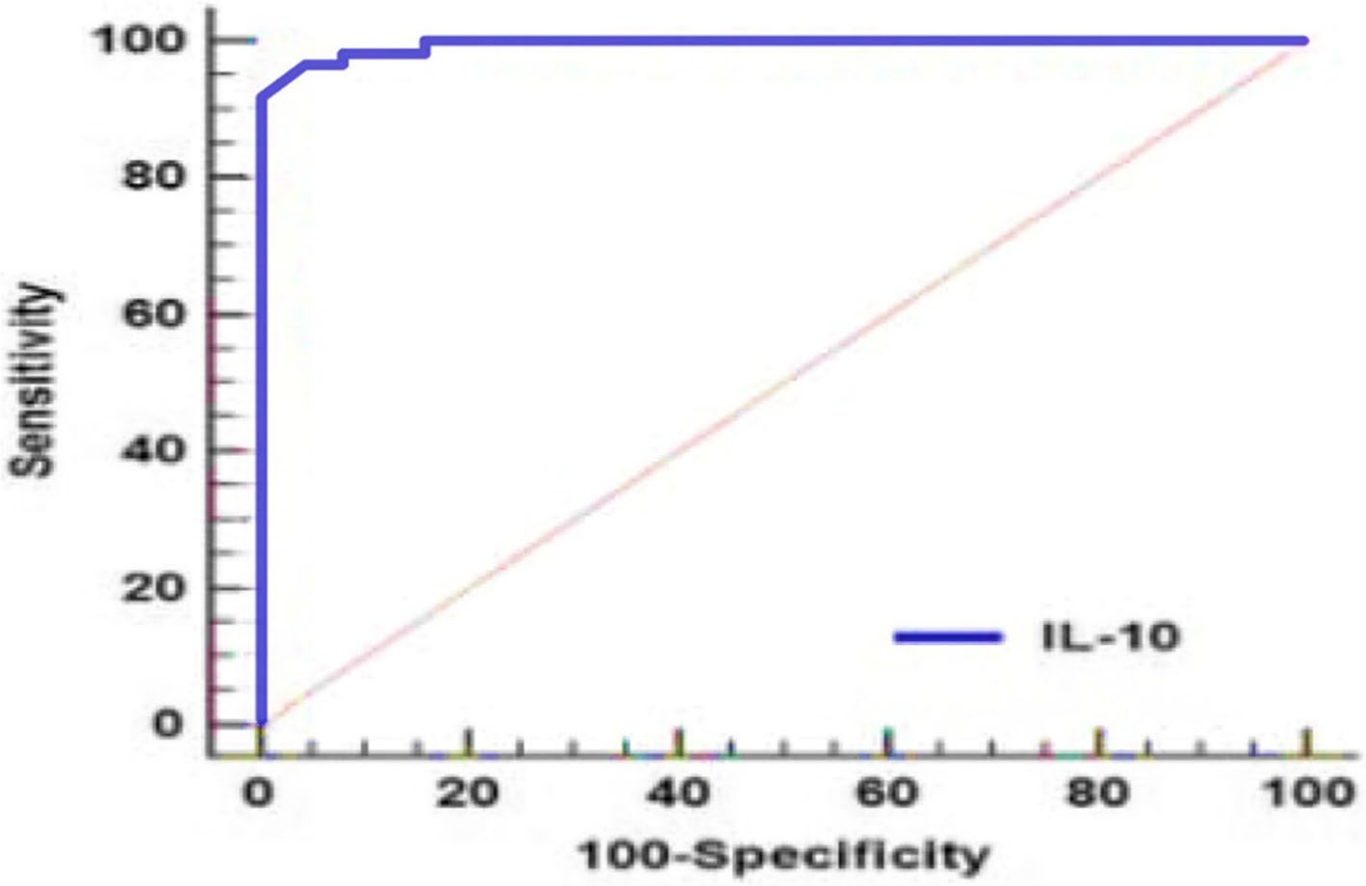
ROC of IL10 for discrimination between ALL cases and control subjects

The validity of IL10 as a diagnostic marker for ALL was assessed in Table 6 & Fig. 8, indicating an area under the curve (AUC) of 0.995 with a 95% confidence interval (CI) of 0.972 to 1. This high AUC suggests excellent discriminatory ability, with an optimal cut-off value of > 361 pg/mL. At this threshold, IL10 demonstrates a sensitivity of 97% and a specificity of 96%, indicating its strong potential as a reliable diagnostic tool for distinguishing ALL cases, with a high sensitivity for detecting true positives and a high specificity for ruling out false positives.

**Table 6:** Validity of IL10 for diagnostic ability of ALL

Variable	AUC	95% CI	Cut off	Sensitivity (%)	Specificity (%)
IL-10	0.995	0.972—1	> 361	97	96

*AUC *Area under ROC curve, *CI *Confidence interval

The receiver operating characteristic (ROC) curve of IL10 was conducted for discrimination between ALL and control groups. IL-10 marker showed high accuracy AUCs (AUC = 0.995). Best cut off value of IL-10 was 361, sensitivity was 97%, specificity was 96.

## Discussion

Acute lymphocytic leukemia is a hematopoietic malignancy characterized by the rapid clonal expansion of immature lymphoid progenitor cells in the bone marrow [6].

Genetic and epigenetic alterations in lymphocyte precursors can lead to continuous proliferation signals and differentiation blockages, resulting in malignancy [19]. Additionally, neoplastic cells can modify the tumor microenvironment to their advantage by producing cytokines and recruiting regulatory cells like Tregs and Myeloid-Derived Suppressor Cells (MDSCs), which aid in evading immune surveillance [20]. One notable mechanism is the promotion of cytokines such as interleukin-10 (IL-10), an anti-inflammatory cytokine from innate and adaptive immune cells, facilitating tumor tolerance [21]. This enables the tumor to evade immune detection by inhibiting inflammatory cytokines like TNF-α and suppressing MHC II, CD80, and CD86 expression.

IL-10, a crucial immunoregulatory cytokine secreted by various lymphoid and myeloid cells, including Th2, CD4, CD8 T cells, NK cells, macrophages, and dendritic cells, can be produced by tumor cells themselves, acting as a cytokine synthesis inhibitory factor (CSIF) leading to immunosuppression [22]. IL-10 suppresses direct T-cell responses by acting on CD4 T cells and inhibits antigen-presenting cells (APCs). Despite inhibiting CD4 T cell responses, IL-10 enhances CD8 T cell proliferation and cytotoxicity. It also suppresses cellular immunity activation and stimulates antitumor responses through interferon-gamma (IFN-γ) and IL-2 secretion, contributing to neoplasm development [23]. This indicates that lymphoproliferation characteristic of cancer cells might stem from a dysfunctional immune system, linking IL-10 to the pathogenesis and prognosis of lymphoid neoplasms [24–26].

The current study demonstrated significantly higher IL-10 levels in ALL patients compared to the control group, consistent with findings by Pérez et al. [27]. Similarly, Nursal et al. [28] observed that tumor cells can diminish natural killer (NK) cells by manipulating their receptors and releasing immunosuppressive factors like IL-10. Conversely, Fitch et al. [29] reported that decreased IL-10 levels accelerated B-cell neoplasms, highlighting the complex role of IL-10 in leukemogenesis and the dysregulated redox landscape in pediatric ALL neoplasms that primes them for leukemic transformation.

Additionally, Kaya et al. [30] and Stevens et al. [31] found elevated levels of IL-10 and migration inhibitory factor (MIF) in pediatric leukemia patients, predicting relapse and poor survival. Saraiva et al. [22] showed that IL-10, secreted by various immune cells, can also be produced by tumor cells to suppress the immune system. Supporting our findings, Fan et al. [32] demonstrated significant associations between IL-10 polymorphisms and susceptibility to various cancers, including ALL. IL-10's role as an anti-inflammatory cytokine involves inhibiting Th1 cytokines like IL-2 and IFN-γ [33], deactivating monocyte/macrophage proinflammatory cytokine synthesis [34], and playing a critical role in immune evasion in leukemia [35].

The multifaceted role of IL-10 in cancer is well-documented, encompassing both tumor-promoting and tumor-suppressive functions [36]. IL-10, a pleiotropic immunoregulatory cytokine secreted by various cells, including lymphoid, myeloid, and tumor cells, mediates immune homeostasis and tolerance while also promoting immunosuppression [22]. It achieves this by inhibiting pro-inflammatory cytokines, downregulating MHC molecules, and promoting regulatory T cell differentiation. This dual role makes IL-10 a double-edged sword: it can suppress anti-tumor immune responses and promote tumor growth, but it also holds potential as a treatment for autoimmune and inflammatory diseases.

Jorgovanovic et al. [23] highlighted the profound implications of IL-10's dichotomous effects on lymphoid cells for cancer immunotherapy. Genetically, IL-10 is located on chromosome 1q31-32, with polymorphic sites in its promoter region that can alter gene transcription and protein function [37, 38]. Studies have linked IL-10 polymorphisms to various cancers, including head and neck, laryngeal, gastric, and hematological neoplasms [24, 38–45].

Specifically, in the context of ALL, studies by Hiroki et al. [37], Li G & Li D [46], and Lo et al. [47] identified the IL-10 gene SNP -1082 G > A (rs1800896) as being associated with increased IL-10 expression and susceptibility to lymphoproliferative disorders, including childhood ALL and lymphoma [24–26, 48]. These findings align with our study, which underscores the role of IL-10 in malignancy predisposition and progression.

Our study revealed a significant association between the *IL 10* rs1800896 polymorphism and ALL in children. Notably, the AG and AA genotypes were linked to a higher risk of ALL in our patient cohort. This finding aligns with Abdalhabib et al. [49], who found a similar association in adult ALL, indicating that the AG and AA genotypes can increase ALL susceptibility, while the overall allele frequency remained similar between patients and controls.

Conversely, Gao et al. [50] reported that the GG genotype of the *IL 10* rs1800896 polymorphism was associated with a decreased risk of leukemia in the general population under a recessive genetic model. This protective effect was particularly evident in non-Chinese individuals, suggesting that genetic and environmental factors interact to influence leukemia risk.

In our study, the A allele of the *IL 10* (G1082A) polymorphism was associated with poor prognosis in Egyptian patients with ALL, corroborating findings by El Baiomy et al. [51]. This highlights the prognostic significance of the *IL 10* polymorphism in ALL.

Additionally, our results showed a cytokine storm in ALL patients, characterized by elevated IL-10 levels, similar to findings by El-Maadawy et al. [52]. This cytokine dysregulation likely contributes to ALL immunopathology, making IL-10 a potential therapeutic target.

We observed significant differences in fever and pallor incidences among rs1800896 genotypes, with the highest incidence in the AA genotype. This suggests that *IL 10* gene polymorphism affects immune response and inflammation, leading to clinical symptoms like fever and pallor. Splenomegaly and lymphadenopathy also varied significantly among genotypes, with the highest incidences in AG and AA genotypes, respectively. These findings indicate that *IL 10* polymorphisms influence pro-inflammatory cytokine production and tissue repair processes.

Our study also found significant differences in CRP levels between rs1800896 genotypes, particularly higher CRP levels in the AG genotype. This suggests that *IL 10* polymorphisms might influence CRP levels by altering the inflammatory balance, with certain polymorphisms leading to increased inflammation.

Overall, our findings emphasize the critical role of *IL 10* gene polymorphisms in influencing ALL susceptibility, clinical manifestations, and prognosis in Egyptian children. Further research in diverse populations is necessary to validate these findings and explore IL-10 as a potential biomarker and therapeutic target in ALL.

Our research highlights the exceptional diagnostic accuracy of IL-10 as a biomarker for ALL, with an area under the curve (AUC) of 0.995. This finding presents exciting opportunities for its application in ALL management. Current screening methods for ALL lack precision, often leading to missed diagnoses or unnecessary investigations.

The IL-10 biomarker stands out as a non-invasive and highly reliable tool, particularly useful in identifying high-risk individuals. By improving screening accuracy, this biomarker addresses the limitations of current methods, reducing the chances of both false negatives and unnecessary diagnostic procedures.

Early detection, facilitated by the IL-10 biomarker, is crucial as it directly affects the timing of intervention. Prompt initiation of treatment, enabled by early detection, can significantly improve prognosis and survival rates in individuals with ALL.

This study not only advances diagnostic precision but also underscores the practical implications of integrating this biomarker into routine clinical practice. Incorporating this high-performing biomarker into ALL screening protocols promises to enhance accuracy, enable timely intervention, and ultimately improve outcomes for individuals at risk of or affected by acute lymphoblastic leukemia.

The present study suggests several recommendations. First, monitoring changes in IL-10 levels and *IL10* − 1082 polymorphism during and after treatment could provide valuable insights into treatment effectiveness and potential relapse risk, enabling timely adjustments to therapy plans. Second, while the findings are promising, larger and more diverse studies are needed to confirm their generalizability and clinical utility. Finally, genetic profiling should be applied to newborns to detect susceptibility to developing ALL early. Combining these biomarkers with established methods, such as genetic testing, could create more powerful diagnostic and prognostic tools.

In conclusion, higher IL-10 levels and the presence of the *IL10* − 1082 polymorphism are independent predictors of ALL susceptibility. Integrating these markers with existing clinical and genetic data could allow for more precise risk stratification of ALL patients. This would enable tailored treatment plans based on individual risk profiles, optimizing outcomes and minimizing unnecessary interventions. Our research underscores the critical role of early detection in the context of ALL. Current screening methods for ALL lack accuracy, leading to missed diagnoses and unnecessary investigations. Introducing novel biomarkers with exceptional sensitivity and specificity presents a promising opportunity to enhance the accuracy of ALL screening.

### Supplementary Information

Supplementary Material 1.

## Data Availability

The datasets used and/or analyzed during the current study are available from the corresponding author on reasonable request.

## Abbreviations

ALL: Acute Lymphoblastic Leukemia
APCs: Antigen-Presenting Cells
CSIF: Cytokine Synthesis Inhibitory Factor
ELISA: Enzyme-Linked Immunosorbent Assay
IFN‐γ: Interferon Gamma
IL: Interleukin
IRB: Institutional Research Board
MDSC: Myeloid Derived Suppressor Cells
MHC: Histocompatibility Complex
MIF: Migration Inhibitory Factor
NCBI: National Center for Biotechnology Information
NK: Natural Killer
REC: Research Ethics Committee
SNP: Single-Nucleotide Polymorphism
TH: T Helper
