# Supplementary material for: Exploring the potential of IL-10 for risk assessment and early intervention in pediatric ALL
Source: BMC Cancer. 2024 Aug 8;24:972. doi: 10.1186/s12885-024-12677-w (PMC11308622; doi:10.1186/s12885-024-12677-w)
Supplement: Supplementary file 1 — Supplementary Material 1. [file 12885_2024_12677_MOESM1_ESM.pdf]

Supplementary Table 1:

S1. Genetic features of studied SNPs according to National Center for Biotechnology Information (NCBI).

ID	***IL10* −1082 A/G**
	**rs1800896**
**Alleles**	AG
**Reference Allele**	A
**Alternative allele**	G
**Gene**	IL10
**Chromosome**	1
**Reference**	***https://www.ncbi.nlm.nih.gov/snp/rs1800896***

The studied SNP, **rs1800896** is composed of A and G alleles, A is the reference and G is the alternative allele, it is located within *IL10* gene, on chromosome 1.

Supplementary Figures 1,2, Full Agarose gel electrophoresis for *IL10* genotypes from different exposures: ladder size marker (M) 50-1000 bp. Where **G** allele at 288bp and **A** allele at 197 bp. 430 bp as internal control. **(Supplementary)**

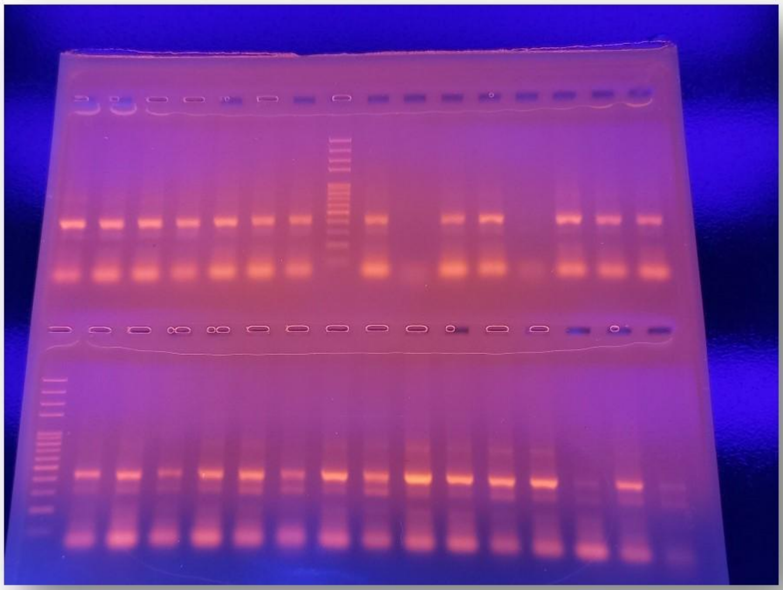

Supplementary Figure 1: S1

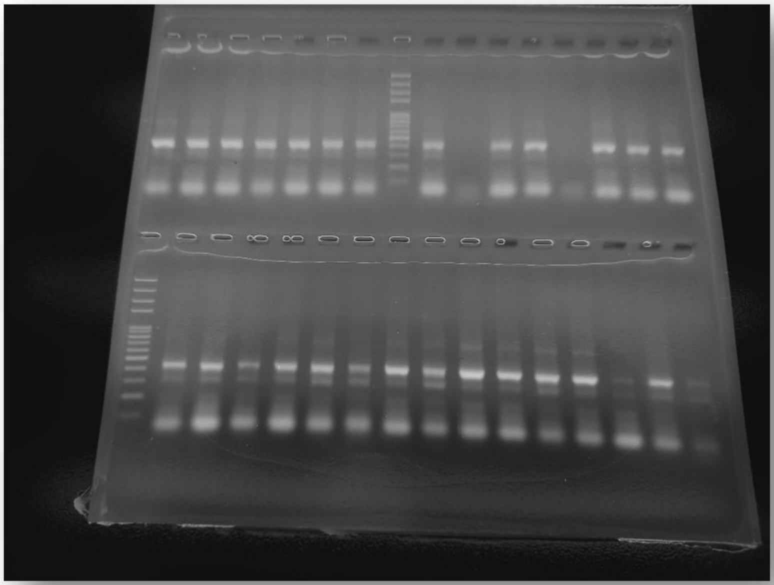

Supplementary Figure 2: S2

Supplementary Table 2:

S2. Association between IL10 −1082 A/G (rs1800896) with clinical presentation among ALL patients.

	IL10 −1082 A/G (rs1800896)						X^2	*p*
	AA n = 13		***AG n = 40***		***GG n = 47***			
Fever								
Absent	0	0.0%	14	35.0%	10	21.3%	9.766	**0.008**
Present	13	100.0%	26	65.0%	37	78.7%		
Pallor								
Absent	1	7.7%	17	42.5%	18	38.3%	6.527	**0.038**
Present	12	92.3%	23	57.5%	29	61.7%		
Fatigue								
Absent	2	15.4%	14	35.0%	15	31.9%	1.997	0.368
Present	11	84.6%	26	65.0%	32	68.1%		
Bleeding Tendency								
Absent	12	92.3%	31	77.5%	36	76.6%	1.940	0.379
Present	1	7.7%	9	22.5%	11	23.4%		

X2, chi square test.

Fever and pallor differed significantly between rs1800896 genotypes, with the highest incidence associated with AA genotype, followed by GG, and AG genotypes (p=0.008, 0.038 respectively). Fatigue and bleeding tendency were not affected by rs1800896 genotypes (p>0.05 for each).

Supplementary Table 3:

S3. Association between IL10 −1082 A/G (rs1800896) with organomegaly among ALL patients.

	IL10 −1082 A/G (rs1800896)						X^2	*p*
	AA n = 13		***AG n = 40***		***GG n = 47***			
Splenomegaly								
Absent	11	84.6%	20	50.0%	32	68.1%	6.312	0.043
Present	2	15.4%	20	50.0%	15	31.9%		
Hepatomegaly								
Absent	3	23.1%	17	42.5%	21	44.7%	2.155	0.340
Present	10	76.9%	23	57.5%	26	55.3%		
Lymphadenopathy								
Absent	1	7.7%	17	42.5%	20	42.6%	7.104	0.029
Present	12	92.3%	23	57.5%	27	57.4%		

X^2, chi square test.

Splenomegaly differed significantly between rs1800896 genotypes, with the highest incidence associated with AG genotype, followed by GG, and AA genotypes (p=0.043). Moreover, lymphadenopathy differed significantly between rs1800896 genotypes, with the highest incidence associated with AA genotype, followed by AG, and GG genotypes (p= 0.038). Hepatomegaly was not affected by rs1800896 genotypes (p>0.05).

Supplementary Table 4:

S4. Serologic data among patients with ALL.

	ALL n = 100	
	Number	*%*
CRP		
Negative	*61*	*61.0*
Positive	*39*	*39.0*

Among all studied ALL cases, 39% had positive CRP and 61% had negative CRP.

Supplementary Table 5:

S5. Association between IL10 −1082 A/G (rs1800896) with CRP among ALL patients.

	IL10 −1082 A/G (rs1800896)			***Test (p1)***	***Post hoc test***		
	AA n = 13	***AG n = 40***	***GG n = 47***		*P2*	*P3*	*P4*
CRP							

Negative	*12 (92.3%)*	*17 (42.5%)*	*32 (68.1%)*	*X2= 12.1, p=0.002*	*0.002*	*0.153*	*0.016*
Positive	*1 (7.7%)*	*23 (57.5%)*	*15 (31.9%)*				

X2, chi square test.

P1: comparison between AA, AG and GG

P2: comparison between AA, AG

P3: comparison between AA, GG

P4: comparison between AG, GG

CRP differed significantly between rs1800896 genotypes (p1=0.002). This was attributed to significant association of positive CRP with AG when compared to AA (p2=0.002), as well as when compared to GG (p4=0.016).

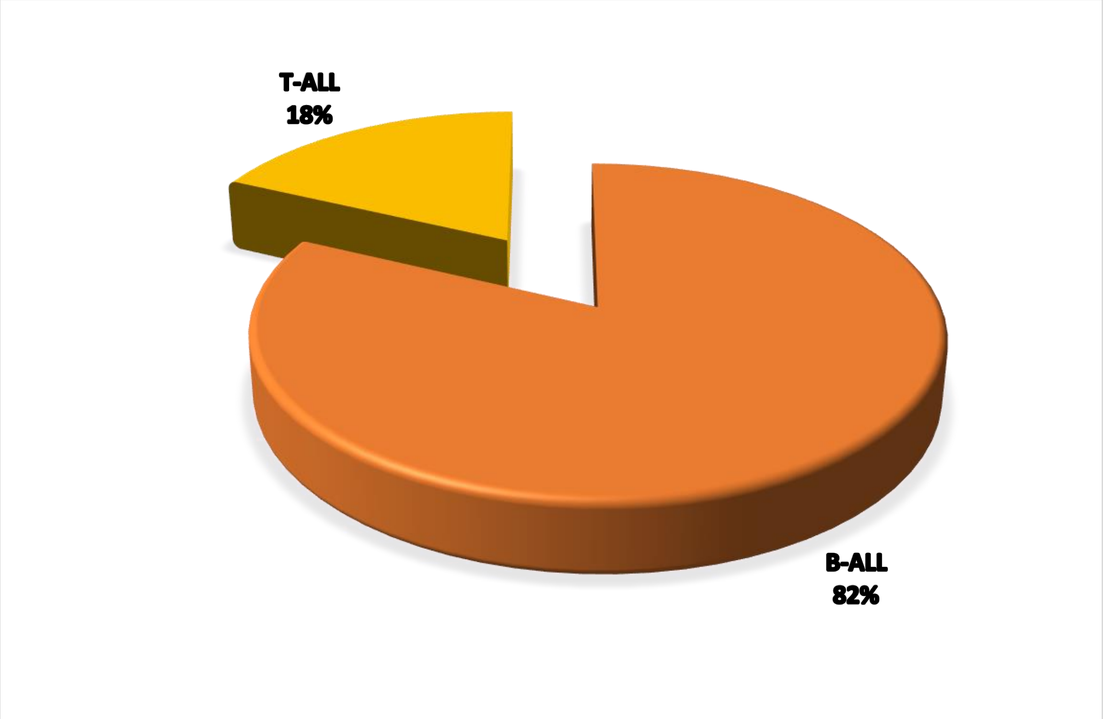

Supplementary Figure 3: S3. Immunophenotyping among patients with ALL

Supplementary Table 6:

S6. Association between IL10 −1082 A/G (rs1800896) with FAB among ALL patients.

	IL10 −1082 A/G (rs1800896)			***Test (p1)***	***Post hoc test***		
	AA ***n = 13***	***AG*** ***n = 40***	***GG*** ***n = 47***		*P2*	*P3*	*P4*
FAB							
L1	*9 (69.2%)*	*31 (77.5%)*	*23 (48.9%)*		*0.712*	*0.194*	*0.006*

L2	*4 (30.8%)*	*9 (22.5%)*	*24 (51.1%)*	*X2= 7.812, p=0.020*			

X2, chi square test.

P1: comparison between AA, AG and GG

P2: comparison between AA, AG

P3: comparison between AA, GG

P4: comparison between AG, GG

FAB classification differed significantly between rs1800896 genotypes (p1=0.020). This was attributed to significant association of L1 with AG when compared to GG, as well as L2 with GG when compared to AG (p4=0.006).
